# Exploring the Combination of Dempster-Shafer Theory and Neural Network for Predicting Trust and Distrust

**DOI:** 10.1155/2016/5403105

**Published:** 2016-01-28

**Authors:** Xin Wang, Ying Wang, Hongbin Sun

**Affiliations:** ^1^School of Computer Technology and Engineering, Changchun Institute of Technology, Changchun 130012, China; ^2^College of Computer Science and Technology, Jilin University, Changchun 130012, China; ^3^Key Laboratory of Symbolic Computation and Knowledge Engineering, Ministry of Education, Changchun 130012, China; ^4^Guangxi Key Laboratory of Trusted Software, Guilin University of Electronic Technology, Guilin 541004, China

## Abstract

In social media, trust and distrust among users are important factors in helping users make decisions, dissect information, and receive recommendations. However, the sparsity and imbalance of social relations bring great difficulties and challenges in predicting trust and distrust. Meanwhile, there are numerous inducing factors to determine trust and distrust relations. The relationship among inducing factors may be dependency, independence, and conflicting. Dempster-Shafer theory and neural network are effective and efficient strategies to deal with these difficulties and challenges. In this paper, we study trust and distrust prediction based on the combination of Dempster-Shafer theory and neural network. We firstly analyze the inducing factors about trust and distrust, namely, homophily, status theory, and emotion tendency. Then, we quantify inducing factors of trust and distrust, take these features as evidences, and construct evidence prototype as input nodes of multilayer neural network. Finally, we propose a framework of predicting trust and distrust which uses multilayer neural network to model the implementing process of Dempster-Shafer theory in different hidden layers, aiming to overcome the disadvantage of Dempster-Shafer theory without optimization method. Experimental results on a real-world dataset demonstrate the effectiveness of the proposed framework.

## 1. Introduction

With the pervasiveness of social media, more and more users participate in various online activities in an unprecedented rate. Trust plays an important role in helping online users make decisions, dissect relevant and reliable information, and receive recommendations. Distrust represents strong negative feelings and insecurity about a user's motivation, intention, and behavior. Fortunately, online social media have made it easy for users to indicate whom they trust and whom they do not. However, trust and distrust relations are often too sparse to be accurately predicted [1]. In addition, the finding in [2] shows that the number of negative links is much smaller compared to positive links. The imbalance and sparsity of social relations bring great difficulties and challenges in predicting trust and distrust.

In essence, constructing trust and distrust relations is a complex cognitive psychology process, which is involved with many theories, such as sociological theory, cognitive theory, and psychological theory. Meanwhile, there are numerous inducing factors to determine trust and distrust relations. However, classical classifiers do not fully take into account the relationship among inducting factors and result in the performance degrading dramatically. The relationship among inducing factors may be dependency, independence, and conflict. Dempster-Shafer theory [3] is an effective and efficient strategy for reasoning with uncertainty, with understood connections to other frameworks such as probability, possibility, and imprecise probability theories. The theory allows combining evidences from different sources and arrives at a degree of belief that takes into account all the available evidences from multiple information sources. While neural network is generally presented as systems of interconnected “neurons” which exchange messages between each other, the connections have numeric weights that can be tuned based on experience, making neural nets adaptive to inputs and capable of learning. Therefore, exploring the combination of Dempster-Shafer theory and neural network can potentially improve the performance and bring new opportunities for trust and distrust prediction.

In this paper, we study predicting trust and distrust based on Dempster-Shafer theory and neural network. In essence, we investigate how to model Dempster-Shafer theory and neural network mathematically and how to incorporate them into predicting trust and distrust. Our contributions are summarized as follows:

- Analyze and quantify inducing factors about trust and distrust, namely, homophily, status theory, and emotion tendency, and verify the research motivations that these inducing factors are contributed to predict trust and distrust.
- Construct evidence prototype based on these inducing factors as input nodes of multilayer neural network, whose advantage is to improve the reliability of evidence features and simplify the complexity of establishing Basic Belief Assignment (BBA) in Dempster-Shafer theory.
- Investigate multilayer neural network to simulate the combining evidences process from different inducing factors in Dempster-Shafer theory, which not only achieves complex decision-making process, but also overcomes the disadvantage of Dempster-Shafer theory without optimization method.

The rest of the paper is organized as follows: Section 2 introduces the related works.  Section 3 depicts the inducing factors about trust and distrust.  Section 4 explores the novel approach for trust and distrust prediction.  Section 5 reports experimental results and performance evaluation. Finally, we summarize our conclusion and suggest possible future directions in Section 6.

## 2. Related Works

### 2.1. Trust Prediction

Trust prediction, which explores unknown relations between online users, is an important research topic in social network analysis. Existing approaches can be grouped into two categories: unsupervised methods and supervised methods.

Most unsupervised methods are based on trust propagation. Trust propagation models focus on developing a trust prediction model which propagates trust values through web of trust. Guha et al. [4] propose a framework of trust propagation schemes that several atomic propagations are introduced, such as direct propagation, cogitation propagation, transpose propagation, and trust coupling propagation, to reduce the sparsity of a web of trust. Kim and Song [5] propose a trust prediction model by discovering reliable trust paths between two users based on reinforcement learning. Oh et al. [6] propose a probability-based trust prediction model based on trust-message passing, which takes advantage of two kinds of information: explicit information and implicit information. However, due to just relying on web of trust, these works based on trust propagation are often too sparse to predict trust with high accuracy.

Supervised methods first extract features from available sources and then construct a classifier based on the labeled trust relations. Zolfaghar and Aghaie [7] first develop a framework of social trust inducing factors and map these factors to corresponding measurable features and then, respectively, apply C5.0 tree and neural net into predicting trust relations. Ma et al. [8] extract various features based on writer-reviewer interaction and apply these features into personalized and cluster-based classification methods. Liu et al. [9] propose a SVM based prediction model to infer the trust relation between two users solely based on their individual actions and interactions in an online community.

Most researches about trust prediction have not paid sufficient attention to the importance of distrust relation [10]. Therefore, links prediction in signed network is becoming an emerging research field.

### 2.2. Links Prediction in Signed Network

Signed network is a special network that creates positive and negative links between nodes. Link prediction in signed network infers new positive and negative links by giving old positive and negative links. Leskovec et al. [2, 11] first apply status theory into better explaining the differences between positive and negative links in online social networks and also use contrasts between balance theory and status theory to draw inferences about how links are being used in particular social computing applications. Yang et al. [12] investigate both unsupervised and semisupervised models to infer signed social ties by capturing the interplay between social relations and users' behavior of decision making and extend the models to encode general principles form social psychology. Hsieh et al. [13] explore the low-rank matrix completion problem to infer sighed networks based on both balance theory and practical points of view. Agrawal et al. [14] propose a more efficient matrix factorization approach for sign inference that recovers the missing links by matrix completion algorithms under certain conditions.

### 2.3. Dempster-Shafer Theory

The Dempster-Shafer theory [3] is a mathematical theory of evidence, which allows one to combine evidences from different sources and arrive at a belief function by taking into account all the available evidences. As a more flexible mathematical tool, Dempster-Shafer theory not only combines with other mathematical frameworks [15–18], but also combines with classifiers [19–21] for dealing with imprecise and uncertain data. In [18], the authors propose an approach that addresses the issue of managing imprecise and vague information in evidential reasoning by combining the Dempster-Shafer theory with the fuzzy set theory. Xiao et al. [15] extend the research of multiple predictions, which use rough set to determine the weight of each single prediction method and utilize Dempster-Shafer theory as the combination method. Basir et al. [19] propose a systematic framework of DSET based on supervised learning for data fusion. They use Dempster-Shafer theory to address the issues of constructing evidence structures and handling dependence between information sources based on neural networks. Denoeux et al. [20, 21] propose an evidential neural network method for pattern classification problems. The assignment of a pattern to a class is made by the degree of support which is defined as a function of the distance between two vectors.

## 3. Data Analysis and Motivating Observations

### 3.1. Data Collection

We take Epinions as experimental dataset which is a famous online social network. Users in Epinions not only write a review by rating items with 1–5 stars, but also rate other reviews using some tags which range from “not helpful” to “most helpful.” In addition to user's behavioral data, the trust and distrust relations are another advantage that users can express their web of trust and block list. Therefore, we first collect the available dataset for this research and pay more attention to direct or indirect social correlations, such as rating the same items, rating other users' reviews, and common neighbours. Then, we delete these users with less than two in-degrees and further filter the users with less than two reviews and ratings, aiming to obtain datasets that are large enough and have sufficient information for the purpose of evaluation.  Table 1 shows some statistics of the collected dataset.

### 3.2. Inducing Factors of Trust and Distrust

In view of sociology and psychology, we divide the inducing factors of trust and distrust into different types of features which include homophily, status theory, and emotion tendency. These features refer to network structures and interaction behaviors in Epinions.

#### 3.2.1. Homophily

Homophily is one of the most important social theories that a contact between similar people occurs at a higher rate than among dissimilar people [22]. We study homophily via the correlation between social relations and users similarity. In this paper, we investigate the following four widely used similarity measures for homophily [23].

*Rating Similarity (RS)*. Rating similarity is equivalent to user's behavior similarity, which can be measured by the cosine similarity of two rating vectors *R*_*i*_ and *R*_*j*_ based on distance. We define rating similarity as

(1) $$\begin{array}{c} \text{RS}\left(\;u_{i},u_{j}\;\right)=\frac{\sum_{k}R_{ik}\cdot R_{jk}}{\sqrt{\sum_{k}R_{ik}^{2}}\sqrt{\sum_{k}R_{jk}^{2}}}. \end{array}$$

*Pearson Correlation Coefficient (PCC)*. Due to different users with different rating styles, namely, some users have the propensity to give higher rating to all items while others probably tend to rate lowly, the similarity of Pearson Correlation Coefficient is defined as

(2) $$\begin{array}{c} \text{PCC}\left(\;u_{i},u_{j}\;\right)=\frac{\sum_{k\in I\left(u_{i}\right)\cap I\left(u_{j}\right)}\left(R_{ik}-\bar{R_{i}}\right)\cdot \left(R_{jk}-\bar{R_{j}}\right)}{\sqrt{\sum_{k}{\left(R_{ik}-\bar{R_{i}}\right)}^{2}}\sqrt{\sum_{k}{\left(R_{jk}-\bar{R_{j}}\right)}^{2}}}, \end{array}$$

where *R*_*ij*_ is the rating to the *j*th item from *u*_*i*_ and *I*(*u*_*i*_) is the set of items *u*_*i*_ rates. $\bar{R_{i}}$ represents the average rate value of *u*_*i*_, and *k* denotes the subset of items rated by both *u*_*i*_ and *u*_*j*_.

*Jaccard's Coefficient (JC)*. To calculate preference similarity between *u*_*i*_ and *u*_*j*_, we model the set of items rated by *u*_*i*_ and *u*_*j*_ as two vectors *I*(*u*_*i*_) and *I*(*u*_*j*_), respectively. Then, we utilize Jaccard's Coefficient [24] to measure the similarity of user preference:

(3) $$\begin{array}{c} \text{Jaccard's Coefficient}\left(\;u_{i},u_{j}\;\right)=\frac{\left|I\left(u_{i}\right)\cap I\left(u_{j}\right)\right|}{\left|I\left(u_{i}\right)\cup I\left(u_{j}\right)\right|}, \end{array}$$

where |·| denotes the size of a set.

*Common Neighbours (CN)*. We exploit user's in-degree and out-degree to measure similarity feature based on common neighbours:

(4) UNui,uj=αOutui∩OutujOutui∪Outuj+1−αInui∩InujInui∪Inuj,

where In(*u*_*i*_) denotes the set of incoming links from other users to *u*_*i*_ and Out(*u*_*i*_) denotes the set of outgoing links from *u*_*i*_ to other users. *α* denotes the weight coefficient of correlation between incoming links and outgoing links.

#### 3.2.2. Status Theory

Status theory is developed to help us understand the important role of social status in the formation of trust and distrust relations. In the context of social networks with graph-based representation, we exploit some measures, such as PageRank, PolarityRank [25], and PolarityIndegree, which are suitable for calculating user social status.

*PolarityRank* is defined as

(5) $$\begin{array}{c} \text{PolarityRank}\left(\;u_{i}\;\right)=\frac{{PR}^{+}\left(u_{i}\right)-{PR}^{-}\left(u_{i}\right)}{{PR}^{+}\left(u_{i}\right)+{PR}^{-}\left(u_{i}\right)}, \end{array}$$

where PR^+^(*u*_*i*_) and PR^−^(*u*_*i*_), respectively, denote the positive PolarityRank and negative PolarityRank of a node *u*_*i*_; both are calculated by (6)

(6) PR+ui=1−αn+α∑j∈In+uiPR+ujOutuj−∑j∈In−uiPR−ujOutuj,PR−ui=1−αn+α∑j∈In+uiPR−ujOutuj−∑j∈In−uiPR+ujOutuj.

*PolarityIndegree.* In the context of trust and distrust, users with larger positive in-degree not only are interpreted as a form of popularity, but also are a symbol of likely enough reliable information source. On the contrary, negative in-degree is also a major factor that leads to a negative impact. Therefore, we employ sigmoid function to measure PolarityIndegree based on positive and negative in-degree:

(7) PI+ui=11+e−αIn+ui−μ,PI−ui=11+e−αIn−ui−μ.

Finally, we normalize PI^+^(*u*_*i*_) and PI^−^(*u*_*i*_) as the final strength of user's social status by

(8) $$\begin{array}{c} \text{PolarityIndegree}\left(\;u_{i}\;\right)=\frac{{PI}^{+}\left(u_{i}\right)-{PI}^{-}\left(u_{i}\right)}{{PI}^{+}\left(u_{i}\right)+{PI}^{-}\left(u_{i}\right)}. \end{array}$$

#### 3.2.3. Emotion Tendency

Social interaction with emotion tendency (ET) is an effective and important way to overcome the problems of imbalance and sparsity of social relations. We explore two features, namely, ET of avg*R*_trustor_ and ET of avg*R*_trustee_, to quantify emotion tendency through comparing user helpfulness ratings with average score of trustor's ratings and trustee's ratings. The helpfulness rating set is represented as *R*_*ij*_ = {*r*_*ij*1_, *r*_*ij*2_,…, *r*_*ijm*_}. We, respectively, define positive emotion tendency ET^+^ and negative emotion tendency ET^−^ by measuring higher and lower than the average rating scores ${\overset{-}{R}}_{trustor}$ and ${\overset{-}{R}}_{trustee}$:

(9) ET+ui,uj=rijk−R−>0Rij,ET−ui,uj=rijk−R−<0Rij,

where *r*_*ijk*_ denotes a helpfulness rating that *u*_*i*_ gives the *k*th review of *u*_*j*_ and $\overset{-}{R}$ denotes the average rating of ${\overset{-}{R}}_{trustor}$ or ${\overset{-}{R}}_{trustee}$.

## 4. Framework of Predicting Trust and Distrust

### 4.1. Problem Statement

**Figure 1:**
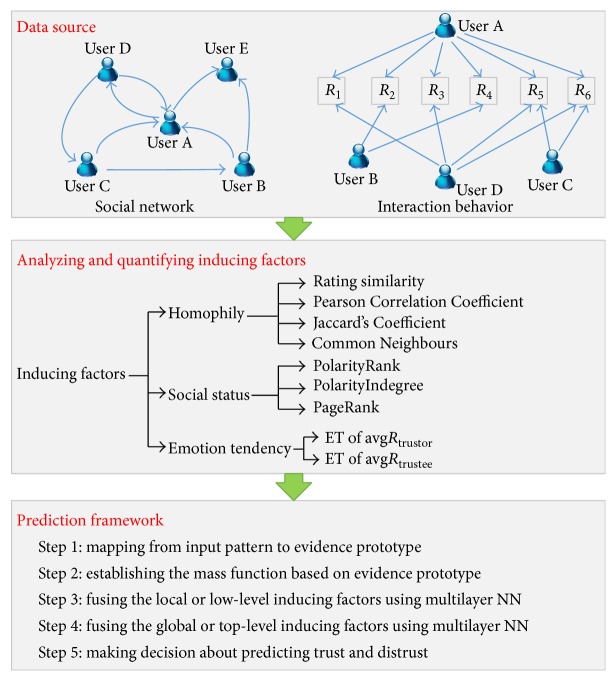
The general framework of predicting trust and distrust.

The problem we study in this paper is to predict trust and distrust based on the binary classification through combining neural network and Dempster-Shafer theory in a supervised way. In this subsection, we first present the notations and then formally define the problem of classification on trust and distrust and depict the general framework in Figure 1.

We use uppercase letter *X* ∈ *R* ^*n*^ to denote an input set *X* = {*x*_1_, *x*_2_,…, *x*_*n*_}, lowercase letter *x* to denote an inducing factor of trust and distrust, and uppercase *C* to denote an output set *C* = {*c*_1_, *c*_2_,…, *c*_*m*_}. The final predicted trust and distrust relations are two class labels, denoted, respectively, as class *c*_1_ and class *c*_2_. In Dempster-Shafer theory, we firstly define a frame of discernment about trust and distrust, which denotes Θ = {trust, distrust}. Then, each inducting factor *x* in input set *X* is mapped to an evidence prototype, which denotes EP. Finally, we construct Basic Belief Assignment (BBA, or called mass function) between evidence prototype EP and target classes *c*, which denotes *m*. In order to overcome the disadvantage of Dempster-Shafer theory without optimization method, we introduce multilayer neural network to dynamically adjust weights in the supervised learning way; all weights are denoted as *ω*.

With the notations above, we formally define classification problem of predicting trust and distrust as follows: given a dataset of all messages with input set *X* of inducing factors and corresponding class labels *C*, on the basis of original features of inducing factors, a series of basic units in our framework are constructed, namely, input unit, evidence processing unit, mass combining unit, fusing unit, and decision unit. Evidence processing unit and fusing unit are, respectively, two smaller multilayer neural networks for handling potential dependence and conflict among evidences and fusing multisource evidences. Finally, all units constitute a larger multilayer neural network as classifier. We aim to learn a classifier to automatically assign class labels for unknown social relations (i.e., test data).

### 4.2. Basic Units of Framework

#### 4.2.1. Input Unit

*Input unit* (IU) achieves the mapping process from initial input set to evidence prototype, where evidence prototype is a representative feature set of quantitative intervals of all inducing factors, denoted as EP = {ep_1_, ep_2_,…, ep_*m*^*n*^_}, where ep_*i*_ denotes the quantitative interval of the *i*th feature. ep_*i*_ is determined by prediction accuracy of trust and distrust in Section 5.2. The power set of evidence prototype with *n* inducing factors and *m* quantitative intervals for each inducing factor has *m* ^*n*^ evidence prototype.

#### 4.2.2. Evidence Processing Unit

*Evidence processing unit* (EPU) is a smaller multilayer neural network with the form of *n* − *h* − 1 for handling potential dependence and conflict among evidences with dynamically adjusting weights in the supervised learning, where *n* denotes the number of input nodes and *h* denotes the number of hidden nodes. Each input node corresponds to a focal element of one evidence source. The output node is the processing result of *n* evidence sources. *ω*_·,*j*_ and *ω*_*j*,·_ are the connection weight set from input node to hidden node *j* and from hidden node *j* to output node, respectively. *f*(·) is the activation function associated with hidden node and output node. At the input-to-hidden and the hidden-to-output layer, the activation function is chosen as logistic sigmoid function, aiming to deal with dependence and conflict between evidence sources through the nonlinear approximation capability of the multilayer neural network. Thus, the activation function of hidden-to-output layer is the processed result of evidence processing unit.

#### 4.2.3. Mass Combining Unit

*Mass combining unit* (MCU) is composed of 2^*c*^ − 1 sum nodes and normalization nodes, where each input node corresponds to the output result of an evidence processing unit; the output node is the normalized combined mass. Due to the requirement of independent sources, we exploit the operation of sum and normalization to achieve Dempster's combination rule on the basis of all evidence processing units. The combination rule of multiple evidences is defined as

(10) $$\begin{array}{c} \overset{n}{\underset{i=1}{⨁}}\;m_{i}\left(\;A\;\right)=\frac{\sum_{A_{1}\cap \cdots \cap A_{n}=A}\prod_{i=1}^{n}m\left(A_{i}\right)}{\sum_{A_{1}\cap \cdots \cap A_{n}\neq Ø}\prod_{i=1}^{n}m\left(A_{i}\right)}. \end{array}$$

#### 4.2.4. Fusing Unit

*Fusing unit* (FU) combines multiple same kind (or level) evidences by using multilayer neural network, which is composed of 2^(*n*+1)^ − 1 evidence processing units and a mass combining unit, where *n* is the number of evidences. In practice, fusing unit can iteratively combine multiple evidences in a hierarchical form, where the local or low-level evidences are fused at the previous stages and the global or top-level evidences are achieved at the final stages. Each fusing unit is trained separately using the outputs from its previous stages.

#### 4.2.5. Decision Unit

*Decision unit* (DU) is used to make the final decision based on the maximum of pignistic probability, where input nodes are the normalized masses of the fusing unit, and output nodes are associated with target class labels. In our framework, DU is applied into the training process of local fusing and the final decision of global fusing.

### 4.3. Framework Architecture

**Figure 2:**
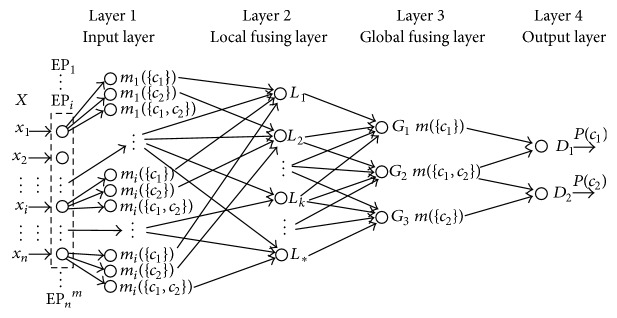
The framework architecture.

The framework architecture includes an input layer, a fusing layer, and a decision layer, respectively, which is shown in Figure 2.

*Layer 1: Input Layer*. We firstly choose the representative features from each type of inducing factor as evidences. Then, we calculate the distance between input set *X* and all evidence prototype and select ep_*i*_ of the minimum distance instead of the initial input set *X* by using

(11) $$\begin{array}{c} {EP}_{i}=\underset{j=1,\ldots ,n^{m}}{\min}\left\{\;\left\|\;X-{ep}_{j}\;\right\|\;\right\}. \end{array}$$

Finally, we establish the Basic Belief Assignment (BBA, or mass function) *m*_*i*_ summarizing the information provided by the decreasing function of distance *ψ*(·) and the degree of class membership *μ*. The monotonically decreasing function *ψ*(*d*_*i*_) of the distance *d*(*x*_*i*_, *I*_ep_*i*__) between the *i*th element *x*_*i*_ in input set and the quantitative interval *I*_ep_*i*__ of the *i*th feature is defined with an exponential form as

(12) $$\begin{array}{c} \psi \left(\;d_{i}\;\right)=\exp\left(\;-d\left(\;x_{i},I_{{ep}_{i}}\;\right)\;\right), \end{array}$$

where the decreasing function *ψ*(0) = 1 and lim_*d*→*∞*_⁡*ψ*(*d*) = 0. The distance function *d*(*x*_*i*_, *I*_ep_*i*__) is defined as |(*a*_max_ − *x*_*i*_)/(*a*_max_ − *a*_min_)|, where *a*_max_ and *a*_min_ are the upper bound and lower bound of quantitative interval of the evidence prototype ep_*i*_, respectively.

In addition, we focus on the correlation between evidence prototype and class membership. We regard class membership as discriminant degree for pattern classification and assume that each evidence prototype is assigned a value *μ*_*q*,*i*_ which represents a degree of class membership about how much feature prototype ep_*i*_ belongs to a class *c*_*q*_. The final *m*_*i*_ can be written as

(13) micq=μq,i·ψdi,miΘ=1−μq,i·ψdi,

where the constraint condition of class membership is ∑_*q*=1_^*M*^ *μ*_*q*,*i*_ = 1. If an evidence prototype fully belongs to a class, it is a special case and is denoted as *μ*_*q*,*i*_ = 1; for other class *k* ≠ *q*, *μ*_*k*,*i*_ = 0.

Thus, each mass function is regarded as the output node *I*_*i*_, which corresponds to a focal element of one evidence source. There are *n* × (2^*c*^ − 1) input nodes in the input layer, where *n* is the number of evidences and *c* is the number of classes.

*Layer 2: Fusing Layer*. We utilize some fusing units to combine multiple evidences in a hierarchical form that begins from local and low-level evidences and then fuses global and top-level evidences. The advantage of fusing unit lies in flexible fusing strategies according to different types of evidences. Therefore, we adopt two strategies of fusing evidences: one is based on the different types of inducing factors; another is according to different types of data attributes, which include network-based and rating-based. The difference of two strategies is that all initial evidences are combined in the local or low-level fusing stage, while both are the same in the global fusing. For the first fusing strategy, three two-source FU modules are applied into combining the same type of inducing factors in local fusing stage. For the second fusing strategy, evidences are fed into two two-source FU for fusing the same type of data attributes in local or low-level fusing stage. The evidence types of two different fusing strategies are shown in Table 2. In global or top-level fusing stage, all evidences obtained from local or low-level fusing stage are combined into the global fusing unit FU simultaneously.

The number of units for two different fusing ways is given in Table 3.

*Layer 3: Decision Layer*. After fusing the local and global evidences, we obtain three mass functions *m*({trust}), *m*({distrust}), and *m*({trust, distrust}). Each output node in this layer is associated with a class. Each node *D* consisted of two incoming masses from fusing layer. The final decision is made by selecting the class with the maximum probability as

(14) $$\begin{array}{c} \omega =\arg\underset{\omega_{n}\in \Theta }{\max}\;D_{n}. \end{array}$$

### 4.4. Parameter Learning

To apply the proposed framework, the parameter learning is required in each FU. The learning process is achieved in a cascading way. In the local or low-level fusing stage, the FU combined with DU is trained by using corresponding training dataset. In the global or top-level fusing stage, each FU is trained based on the estimated masses, which are transferred from the best outputs of FUs in the local or low-level stage.

Each FU in the fusing layer has similar parameters and learning procedure. Let us take any FU as an example. *ω*_·,*j*_ and *ω*_*j*,·_ are the connection weight set from input node to hidden node *j* and from hidden node *j* to output node, respectively. Each hidden node performs the weighted sum of its inputs to form its net activation, net_*j*_, which is defined as a formation of inner product of the inputs with the weights at the hidden node:

(15) $$\begin{array}{c} {net}_{j}=\overset{d}{\underset{i=1}{\sum }}m_{i}\omega_{ji}+\omega_{j0}=\overset{d}{\underset{i=i}{\sum }}m_{i}\omega_{ji}={w^{t}}_{j}m, \end{array}$$

where *m*_*i*_ denotes the *i*th mass function on the input layer. *ω*_*ji*_ denotes the weight of hidden node *j* of input-to-hidden layer. **w** and **m** are the vector formation of both. The weight settings are contributed to improve prediction accuracy and fast converge of iterative algorithm. The initial weights of **w** are set according to the feature effectiveness of inducing factors in our experiments. Each hidden node emits an output that is a nonlinear function of its activation *f*(·):

(16) $$\begin{array}{c} y_{j}=f\left(\;{net}_{j}\;\right), \end{array}$$

where we choose a logistic sigmoid function as nonlinear activation function.

Each output node also calculates its net activation in the hidden-to-output layer:

(17) $$\begin{array}{c} {net}_{k}=\overset{h}{\underset{i=1}{\sum }}y_{j}\omega_{kj}+\omega_{k0}=\overset{h}{\underset{i=i}{\sum }}y_{j}\omega_{ki}={w^{t}}_{k}y, \end{array}$$

where *k* denotes the index of node in the output layer and *h* denotes the number of hidden nodes, and *y*_*j*_ = *f*(net_*j*_).

Similar to the hidden node, each output node is calculated by using the nonlinear activation function:

(18) $$\begin{array}{c} z_{k}=f\left(\;{net}_{k}\;\right). \end{array}$$

The learning parameters are estimated and adjusted by using a multilayer neural network with least squared error function. The error function is based on minimizing the sum of squared difference between the actual output *z*_*k*_ and the desired output *t*_*k*_, which is defined as

(19) minω Jω=12∑k=1ctk−zk2,

where *ω* denotes all the weights in the multilayer neural network. To avoid overfitting, we add two smoothness regularizations on weight *ω*_·,*j*_ and weight *ω*_*j*,·_; then we have

(20) minω·,j,ωj,· Jω·,j,ωj,·=12∑k=1ctk−zk2+α2∑iω·,j2+β2∑kωj,·2,

where *α* and *β* are the regularization parameters which trade off the error function cost with the larger weights penalization.

Then, we derive the back propagation algorithm that is based on gradient descent of error function on the weight *ω*_·,*j*_ and *ω*_*j*,·_ is given by

(21) ω·,j⟵ω·,j−η∂J∂ω·,j−ηαω·,j,ωj,·⟵ωj,·−η∂J∂ωj,·−ηβωj,·,

where *η* is the learning rate and merely determines how much an updating step influences the current weights. The new terms *ηαω*_·,*j*_ and *ηβω*_*j*,·_ cause the weights to decay in proportion to its size when the weights are updated in the process of iteration.

Next, we turn to the problem of evaluating ∂*J*/∂*ω*_·,*j*_ and ∂*J*/∂*ω*_*j*,·_ on (21). As the operations in MCU and DU are fixed without change, the weight changing is only limited to EPU with multilayer neural network. Thus, we can apply the chain rule to obtain partial derivatives of the error function with respect to the weights *ω*_·,*j*_ and *ω*_*j*,·_:

(22) ∂J∂ω·,j∂J∂zk∂zk∂netk∂netk∂ω·,j=tk−zkf′netkyj,∂J∂ωj,·∂J∂yj∂yj∂netj∂netj∂ωj,·=xif′netj∑kωkjtk−zkf′netk.

## 5. Experiments

### 5.1. Experimental Settings and Evaluation Metric

The experimental settings of trust and distrust prediction are described as follows: we firstly filter some user pairs, such as the similarity that is equal to zero in homophily and users without any helpfulness ratings in emotion tendency. Then, we adopt the Guha methodology [4] and construct a balance dataset with equal numbers of trust and distrust predictions. Finally, let *A* = {〈*u*_*i*_, *u*_*j*_〉∣*G*(*i*, *j*) = 1, or − 1} be the set of user pairs with trust and distrust. We randomly divide set *A* into two parts *L* and *N*. *x*% of *A* denoted as *L* is chosen as the training dataset. The remaining 1 − *x*% of *A* denoted as *N* is designated for the testing dataset, whose signs of relations are hidden by setting *G*(*i*, *j*) = 0.

We follow the common metric to evaluate the effectiveness of inducing factors and the proposed framework. In detail, we take the signs of relations obtained by learning model as the set of predicted results, denoted as *R*. Then, the prediction accuracy (PA) [2] is defined as |*N*∩*R*|/|*N*|, where |·| denotes the size of a set. We conduct all experiments 5 times to ensure that our results are reliable and average the result on the evaluation metric.

### 5.2. Feature Effectiveness of Inducing Factors

**Figure 3:**
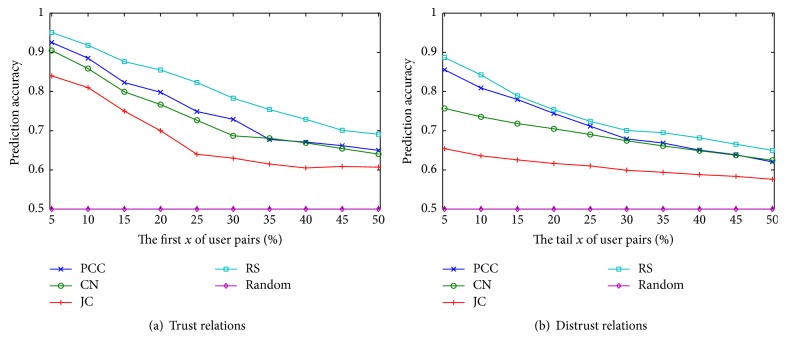
Prediction accuracy of trust and distrust about homophily.

**Figure 4:**
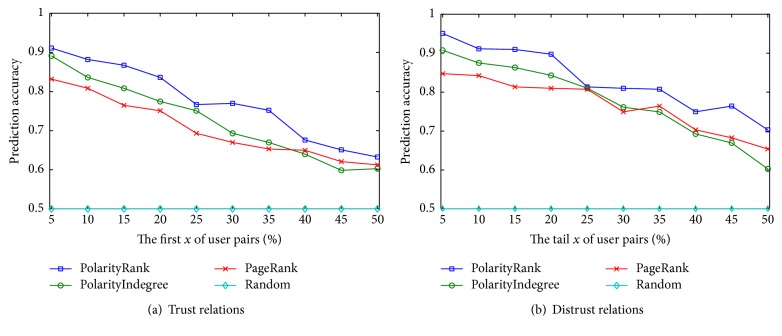
Prediction accuracy of trust and distrust about social status.

**Figure 5:**
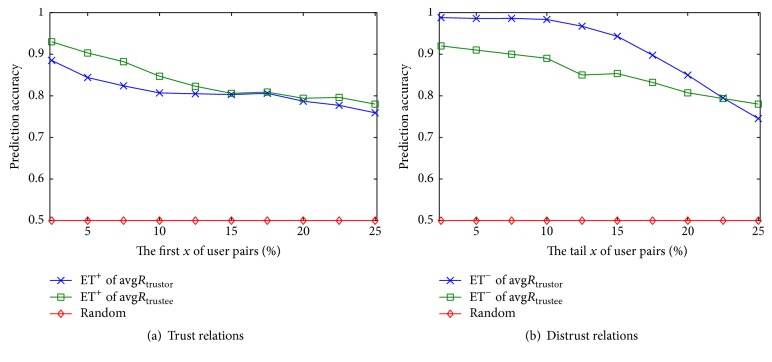
Prediction accuracy of trust and distrust relations about emotion tendency.

We rank all user pairs in *A* in a descending order based on feature weights of inducing factors and take the first *x*% of user pairs as trust relations and take the tail *x*% of user pairs as distrust relations. *x* is varied as {5,10,15,20,25,30,35,40,45,50}. We compare the predicted results with the original signs of relations to measure the prediction accuracy. For comparison, we set a random guessing program as a baseline method. All the features of inducing factors help to construct mass functions and initialize the weights of hidden nodes in local fusing state. The evaluated results are illustrated in Figures 3–5, respectively. We draw the following observations.

(*1) Homophily*. In Figures 3(a) and 3(b), RS obtains the best performance among the four measures of homophily coefficient. We also note that RS, PCC always obtain better performance than CN, JC, and random. It suggests that helpfulness rating is more effective than network topology in predicting social relations. Meanwhile, since those user pairs with distrust relations also have a certain degree of similarity, the performance of trust prediction is superior to distrust prediction for homophily.

 (*2) Social Status*. In Figures 4(a) and 4(b), the effectiveness of predicting trust relations is worse than distrust relations for social status. The main reason is that users with high statuses are more active; the number of their constructing trust and distrust relations is greatly more than that of users with low statuses. We also note that the performance of PolarityRank and PolarityIndegree is very similar, which is much better than that of PageRank.

 (*3) Emotion Tendency*. In Figures 5(a) and 5(b), since users are likely to give positive ratings (the majority of ratings are with 4 or 5 stars), predicting trust relations based on emotion tendency leads to a bit of confusion and has a certain degree of error rate. However, negative emotion tendency is obviously more discriminated than positive one; the effectiveness of predicting distrust relations is relatively clear. In addition, the performance of ET of avg*R*_trustor_ is better than ET of avg*R*_trustee_ in terms of trust and distrust prediction.

**Figure 6:**
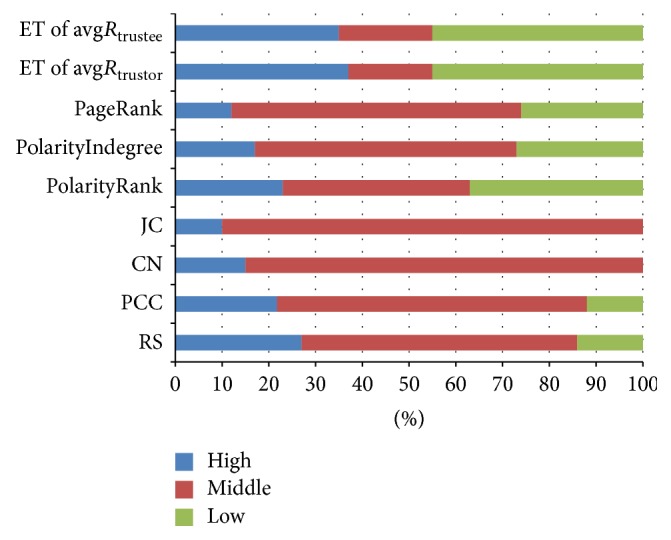
The discretization result of evidence prototype.

On the whole, with the increase of *x*, feature effectiveness of inducing factors reduces, however, still much better than that of random guessing. It is consistent with the previous observations that the task of predicting trust and distrust is more obvious at first and tail region; on the contrary, trust and distrust are confused and difficult at middle region. In addition to comparing the effectiveness of different inducing factors, our other objective is to discrete features of inducing factors and to determine the quantitative interval of evidence prototype in our proposed framework. We stratify features of inducing factors into *m* levels according to the acceptable results of prediction accuracy. In our experiments, we choose *m* = 3 for all cases and divide feature strength into three levels, such as high, medium, and low, respectively. The discretization result of feature prototype is shown in Figure 6, if the acceptable prediction accuracy is assigned as 80%, which we can get the order of feature effectiveness. For trust relations, the order of feature effectiveness is ET of avg*R*_trustor_, ET of avg*R*_trustee_, RS, PCC, PolarityRank, PolarityIndegree, and so on. For distrust relations, ET of avg*R*_trustor_, ET of avg*R*_trustee_, PolarityRank, and PolarityIndegree are several more effect features. In local fusing state, the weight settings of hidden nodes in EPU are based on the rank of feature effectiveness.

### 5.3. Impact in Different Types of Evidences

On the basis of analyzing all features effectiveness, we use the logistic regression method of LIBLINEAR (http://www.csie.ntu.edu.tw/~cjlin/liblinear/) to evaluate the impact in different types of evidences. The evaluated results help to initialize weight settings of hidden nodes in global fusing state. In detail, we compare two fusing ways to evaluate the impact in different types of evidences: one way is based on the different types of inducing factors; another one is based on the different types of data sources, such as network structure and interaction behaviors. In two groups of experiments, we vary the percentage of labeled relations from 10% to 50% for training.

Table 4 lists the classification performance on different inducing factors. We can see that emotion tendency achieves the highest performance, which is 0.845 at the 30% labeled relations and gradually stabilizes. The performance on homophily is even slightly worse than that of other methods. It is consistent with our previous observations that homophily does not have good discriminating ability regarding trust and distrust.

We, respectively, integrate the features of CN and PolarityRank into network structure and the features of RS and emotion tendency into interaction behaviors. The classification performance of two different types of data sources is shown in Table 5. We can see that the classification method achieves better performance when using interaction behaviors compared to network structure data in terms of prediction accuracy. Specifically, the performance with interaction behaviors improves the prediction accuracy by 0.05–0.12 compared to the network structure. In addition, from both Tables 4 and 5, the fusing way based on inducing factors outperforms the fusing way of data sources.

### 5.4. Performance of the Proposed Framework

In order to demonstrate the effectiveness of the proposed framework, we compare with several baseline methods:

- *Random*: a random guessing result yields 50% prediction accuracy, since trust and distrust in dataset are balanced.
- *SVM*: we apply SVM classifier on the features of inducing factors, which is similar to the logistic regression model.
- *DT*: C5.0 decision tree is constructed by exploiting information gain on features of inducing factors.
- *NN*: we regard neural network with multilayer as the multisources data fusion tool, where input nodes are corresponding to feature of inducing factors.
- *DS*: previous work [26] applies Dempster-Shafer theory on Epinions to predict trust and distrust, which only includes rating data, without a web of trust.
- *CDN*: our proposed approach combines Dempster-Shafer theory and neural network to predict trust and distrust.

As the sparsity of social relations and interaction data, we only use 40% of the labeled data for training.  Table 6 shows the performance comparison of the different classification methods. Our approach, CDN, gives 0.067–0.113 improvements on prediction accuracy over DT and SVM methods. When all features of inducing factors are integrated into the SVM classifier, there is no significant improvement of performance compared with the previous logistic regression. In addition, since feature ranking technique is applied into the C5.0 decision tree, the performance of DT is better than SVM (+0.046).

Table 7 lists the performance comparison of the different methods for data fusion technique. It is clear that our proposed framework CDN significantly outperforms the other baseline methods. Specifically, CDN improves the prediction accuracy by 0.045–0.079 compared with the NN and DS methods. From both Tables 6 and 7, we can arrive at a conclusion that our approach can clearly improve performance on predicting trust and distrust.

## 6. Conclusion and Future Work

In this paper, we firstly analyze and quantify inducing factors about trust and distrust and verify the research motivations that these inducing factors are contributed to predict trust and distrust relations. Then, we devise the evidence prototype based on these inducing factors, which improve the reliability of evidence features and simplify the complexity of establishing the Basic Belief Assignment in Dempster-Shafer theory. Finally, we propose a framework to predict trust and distrust based on combining Dempster-Shafer theory and neural network, which investigate a multilayer neural network to model the implementing process of Dempster-Shafer theory of evidence in different hidden layers, aiming to overcome the disadvantage of Dempster-Shafer theory without optimization method. Experimental results on a real-world dataset demonstrate the effectiveness of the proposed framework.

There are several directions needing further investigation. Firstly, we will incorporate our framework to other social media applications, such as tie strength prediction, sign prediction, and recommendation system. Secondly, we plan to explore new models and algorithms on predicting trust and distrust, such as sparse learning and deep learning.

## Figures and Tables

**Table 1:** Statistics of the dataset.

	Epinions
# of users	9718
# of reviews	646201
# of ratings	9160113
# of relations	394595
# of trust relations	330671
# of distrust relations	63924

**Table 2:** The evidence types of two different fusing strategies.

Fusing strategies	Evidence type	Feature name
Inducing factors	Homophily Social status Emotion tendency	RS, PCC PolarityRank, PolarityIndegree ET of avg*R*_trustor_, ET of avg *R*_trustee_

Data attributes	Network-based Rating-based	CN, PolarityRank RS, ET of avg*R*_trustor_

**Table 3:** The number of units for two different fusing strategies.

Fusing strategies	# of units at local or low-level fusing			# of units at global or top-level fusing		
	FU	EPU	MCU	FU	EPU	MCU
Inducing factors	3	21	3	1	15	1
Data attributes	2	14	2	1	7	1

**Table 4:** Classification performance of different types of inducing factors.

Type of inducing factor	Percentage of labeled relations				
	10%	20%	30%	40%	50%
Homophily	0.542	0.652	0.705	**0.746**	0.745
Social status	0.585	0.702	0.726	**0.766**	0.764
Emotion tendency	0.679	0.785	**0.845**	0.84	0.843

**Table 5:** Classification performance of different types of data sources.

Type of data source	Percentage of labeled relations				
	10%	20%	30%	40%	50%
Network structure	0.537	0.650	0.721	**0.763**	0.760
Interaction behavior	0.652	0.724	**0.813**	0.810	0.812

**Table 6:** Performance comparison of different classification methods.

Evaluation metric	Classifier technique			
	Random	SVM	DT	CDN
PA	0.50	0.801	0.847	**0.914**

**Table 7:** Performance comparison of different fusion methods.

Evaluation metric	Data fusion methods		
	NN	DS	CDN
PA	0.869	0.835	**0.914**
